# The effects of the chemical environment of menaquinones in lipid monolayers on mercury electrodes on the thermodynamics and kinetics of their electrochemistry

**DOI:** 10.1007/s00249-021-01512-w

**Published:** 2021-03-17

**Authors:** Karuppasamy Dharmaraj, Dirk Dattler, Heike Kahlert, Uwe Lendeckel, Felix Nagel, Mihaela Delcea, Fritz Scholz

**Affiliations:** 1 Institute of Biochemistry, University of Greifswald, Felix-Hausdorff-Str. 4, 17487 Greifswald, Germany; 2 Institute of Medical Biochemistry and Molecular Biology, University Medicine Greifswald, University of Greifswald, Ferdinand-Sauerbruch-Str, 17475 Greifswald, Germany

**Keywords:** Menaquinone, Vitamin K_2_, Electrochemistry, Thermodynamics, Kinetics, Lipid monolayers

## Abstract

The effects of the chemical environment of menaquinones (all-*trans* MK-4 and all*-trans* MK-7) incorporated in lipid monolayers on mercury electrodes have been studied with respect to the thermodynamics and kinetics of their electrochemistry. The chemical environment relates to the composition of lipid films as well as the adjacent aqueous phase. It could be shown that the addition of all-*trans* MK-4 to TMCL does not change the phase transition temperatures of TMCL. In case of DMPC monolayers, the presence of cholesterol has no effect on the thermodynamics (formal redox potentials) of all-*trans* MK-7, but the kinetics are affected. Addition of an inert electrolyte (sodium perchlorate; change of ionic strength) to the aqueous phase shifts the redox potentials of all-*trans* MK-7 only slightly. The formal redox potentials of all-*trans* MK-4 were determined in TMCL and nCL monolayers and found to be higher in nCL monolayers than in TMCL monolayers. The apparent electron transfer rate constants, transfer coefficients and activation energies of all-*trans* MK-4 in cardiolipins have been also determined. Most surprisingly, the apparent electron transfer rate constants of all-*trans* MK-4 exhibit an opposite pH dependence for TMCL and nCL films: the rate constants increase in TMCL films with increasing pH, but in nCL films they increase with decreasing pH. This study is a contribution to understand environmental effects on the redox properties of membrane bond redox systems.

**Supplementary Information:**

The online version contains supplementary material available at 10.1007/s00249-021-01512-w.

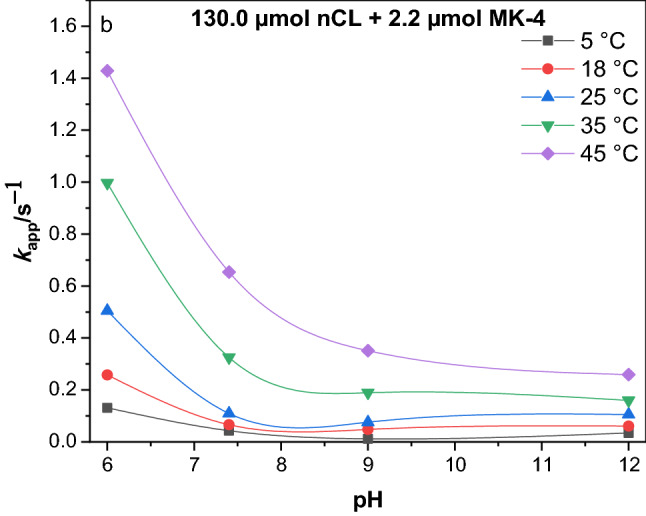

## Introduction

Menaquinones (MK-*n*), the vitamin K_2_ class of compounds with a 2-methyl-1,4-naphthoquinone moiety connected with *n* isoprenyl units, are crucially involved in diverse biological functions and insufficient levels of vitamin K result in diseases (Schurgers and Vermeer 2002; Halder et al. 2019; Sato et al. 2020; Farhadi Moghadam and Fereidoni 2020). Indeed, only the all*-trans* form of MK-7 is biological active (Lal et al. 2020). Recently, the acid–base and the redox properties of all*-trans* MK-4, -7, and -9 in 1, 2-dimyristoyl-sn-glycero-3-phosphocholine (DMPC) monolayers on mercury electrodes have been studied (Dharmaraj et al. 2020). This has been done because only very limited electrochemical data were available (Lovander et al. 2018), particularly for vitamins K in biological membranes. Composition effects, including the nature of lipid phases, cholesterol content, and inert salt addition to the aqueous phase, and also temperature effects on the redox properties of menaquinones in membranes are of interest to understand the complex membrane machineries. For instance, phase transitions of the lipids in membranes are known to have a strong effect on the permeation of H^+^/OH^−^ ions (Elamrani et al. 1983). Model systems, such as lipid monolayers and liposomes, can be used to understand the thermodynamics and kinetics of the redox reactions. Lipid monolayers on mercury electrodes are excellent model systems because the measurements are highly reproducible, among others, because the formation and structure of the monolayers on mercury are highly reproducible. Important questions to be addressed are: (i) how does the nature of lipids affect the redox potential of quinoid membrane constituents (e.g., of ubiquinone (Heise et al. 2017), menaquinones, etc.)? (ii) How does the cholesterol content of the membranes affect the redox properties of quinoid membrane constituents (Schroeder et al. 1991), and how it affects the membrane fluidity, ion transport, signal transduction, etc. (Simons et al. 2004; Levitan et al. 2010; Bastiaanse et al. 1997; Fielding et al. 2004; Lange et al. 2016; Madden et al.^1980^; Cornelius 2001)? (iii) How does the addition of an inert salt affect the redox properties of the quinoid membrane constituents? Inert salts change not only the ionic strength, but also the water activity, which is known to have an effect on the intramolecular properties at catalytic sites (Disalvo 2015; George et al. 1970). Here, we report attempts to partially answer these questions by experiments in which menaquinones have been incorporated in lipid monolayers on a stationary mercury drop electrode. This approach allows analyzing both the thermodynamics as well as the kinetics of electrochemistry of the naphthoquinone/naphthohydroquinone redox couple. The results may allow drawing conclusions with respect to the chemical redox switching when the menaquinones operate in the respiration chain.

## Experimental section

### Chemicals

The following chemicals were used: trisodium citrate pentahydrate (extra pure) and sodium perchlorate (NaClO_4_) (extra pure) were from Laborchemie, Apolda GmbH, Germany; Disodium monohydrogen phosphate dihydrate (Na_2_HPO_4_·2H_2_O) (≥ 98%), sodium hydroxide (NaOH) (≥ 99%), potassium chloride (KCl) (≥ 99.5%), chloroform (CHCl_3_) (HPLC grade) and methanol (CH_3_OH) (≥ 99.98%, ultra LC–MS grade) were from Carl Roth GmbH, Germany; monosodium dihydrogen phosphate dihydrate (NaH_2_PO_4_·2H_2_O) (pure pharma grade) was from Applichem GmbH, Germany; disodium carbonate monohydrate (Na_2_CO_3_·H_2_O) (> 99%) was from Fluka Chemika, Germany; mercury (99.9999 Suprapur), hydrochloric acid (HCl) ((32%) for analysis), sodium bicarbonate (NaHCO_3_) and citric acid monohydrate (analytical grade) were from Merck, Germany; DMPC (14:0 PC) (1,2-dimyristoyl-sn-glycero3-phosphocholine) (> 99%), TMCL (1,1′,2,2′ tetramyristoyl cardiolipin) (14:0 cardiolipin (sodium salt)) (1′,3′-bis[1,2-dimyristoyl-sn-glycero-3-phospho]-glycerol (sodium salt)) (> 99%) and nCL (cardiolipin (bovine heart) (sodium salt)) (> 99%) lipids were from Avanti Polar Lipids, USA; all*-trans* menaquinone 4 (all*-trans* MK-4) (analytical standard), all*-trans* menaquinone 7 (all*-trans* MK-7) (United States Pharmacopeia (USP) Reference Standard) and cholesterol (Sigma Grade ≥ 99%) were from Sigma-Aldrich, Germany. The buffer solutions were prepared using citric acid monohydrate/trisodium citrate pentahydrate for pH 4.0, Na_2_HPO_4_·2H_2_O/NaH_2_PO_4_·2H_2_O for pH 6.0 and 7.4, Na_2_CO_3_·H_2_O/NaHCO_3_ for pH around 9.0, and NaOH for pH 12.0 (Dawson et al. 1986). For adjusting the buffer pH, HCl and NaOH were used.

### Instrumentation

The electrochemical measurements were performed with the AUTOLAB PGSTAT 12, in conjunction with the electrode stand VA 663 (Metrohm, Switzerland). A multimode electrode in which the hanging mercury drop electrode (HMDE mode) (drop size 2, surface area 0.464 mm^2^) served as working electrode, a platinum rod and an Ag | AgCl (3 M KCl, *E* = 0.207 V vs. SHE (standard hydrogen electrode)) (connected to the cell via a saturated KCl salt bridge) electrode were used as auxiliary and reference electrodes, respectively. The redox systems were studied with cyclic voltammetry (staircase) in normal mode applying different scan rates with step potential of 0.00045 V. A temperature-controlled bath (Lauda Ecoline 003 E100) was used for all measurements. The calorimetric measurements were recorded with a MicroCal VP-DSC by Malvern Panalytical at the scan rate of 90 K/h.

### Liposome preparation

The liposomes were prepared according to Moscho’s rapid evaporation technique (Moscho et al. 1996). The lipids (DMPC, TMCL and nCL), cholesterol, and the MK were dissolved separately in chloroform to prepare stock solutions. The lipids from the stock solution were diluted with chloroform and methanol (ratio 3:1) and the desired amount of menaquinone was added from the chloroform stock solution (1 mg mL^−1^), so that the desired molar ratio lipid:menaquinone (60:1) was reached. This was followed by adding 20 mL of aqueous buffer (pH 7.4). The organic solvents were removed using the rotation evaporator Laborota 4000 (Heidolph, Germany) and the Rotavac control pump (Heidolph, Germany) at 50 °C, 60 rpm and a final pressure of 100 mbar. For the liposomes containing cholesterol (Hernández et al. 2008a, 2008b), the desired amount of lipids, cholesterol and all*-trans* MK-7 were diluted with chloroform and methanol (ratio 3:1) in a round bottomed flask and the solvents were removed at 45 °C and a final pressure of 100 mbar. After the solvent evaporation, the lipid–cholesterol–all*-trans* MK-7 film was dried again with a stream of nitrogen for 30 min. The aqueous buffer pH 7.4 (30 mL) was added into the round bottomed flask with glass pearls containing the dried films on the inner side of the glass vessel and kept in the water bath (45 °C) at 180 rpm for 10 min. The hydrated liposome suspension was extruded at 45 °C with a total of 10 passes through a 400 nm filter using the Avanti Mini Extruder (Avanti Polar Lipids, Inc., USA). The total amount of DMPC or DMPC/Chol composition was 300 µmol.

### Electrochemical measurements

The melting point (*T*_m_) of DMPC is 23.9 °C (Mabrey et al. 1976). Three phase transition regions have been found in the DMPC–cholesterol system: existence of gel (G) or fluid lamellar disordered phases (L_α_ (d)) at low cholesterol (~ < 6 mol %) content, fluid lamellar ordered (L_α_ (o)) phases at high cholesterol content (~ > 30 mol %) and between these, the existence of G + L_α_ (o) or L_α_ (d) +  L_α_ (o) phases (Almeida et al. 1992; Hernández et al. 2008a, 2008b). Therefore, three DMPC/Chol compositions 95/5 mol%, 80/20 mol% and 65/35 mol% at 20 °C and 28 °C temperatures were chosen for the electrochemical investigations. 5 µmol all*-trans* MK-7 was used for the studies of cholesterol and water activity on all*-trans* MK-7 measurements. Sodium perchlorate was used to interrogate the effect of an inert salt, and thus, also for the effect of water activity at 25 °C in aqueous buffer pH 7.4. The TMCL (1′,3′-bis[1,2-dimyristoyl-sn-glycero-3-phospho]-glycerol (sodium salt)) exhibits the lamellar gel ( L_β_) to lamellar liquid crystalline (L_α_) and subgel (L_c_) to lamellar gel (L_β_) transitions at 40.3 °C and 24.2 °C, respectively. Addition of 2.2 µmol all*-trans* MK-4 to 130 µmol TMCL has practically no effect on transition temperatures (40.7 °C and 23.8 °C) (Fig. S1). Natural cardiolipins (nCL) and nCL containing all*-trans* MK-4 liposomes do not exhibit any phase transitions in the temperature range 7–90 °C. The voltammetric measurements to study the behavior of all*-trans* MK-4 in different cardiolipin phases were performed at 5 °C, 18 °C, 25 °C, 35 °C, and 45 °C. A non-isothermal electrochemical cell configuration was used by keeping the reference electrode at ambient temperature. The liposome suspension was deaerated for at least 30 min. A mercury drop was formed and the solution was stirred for 15 min to form a monolayer. The liposome solution was replaced with aqueous buffer, and the buffer solution was purged with nitrogen to remove the dissolved oxygen. Then, the monolayer was characterized by electrochemical measurements.

## Results and discussion

### Thermodynamics of the electrochemistry of menaquinones in DMPC/cholesterol monolayers on mercury

**Fig. 1:**
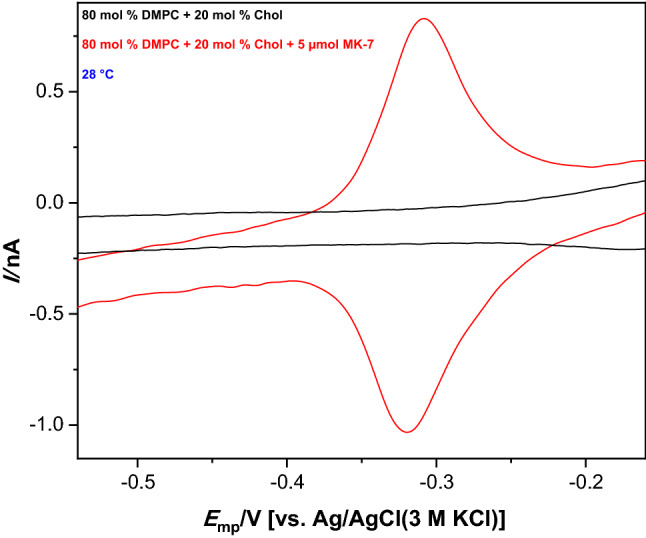
Cyclic voltammograms of DMPC/Chol and DMPC/Chol/all*-trans* MK-7 films in pH 7.4 at 28 °C. Scan rate: 10 mV s^−1^

In DMPC/Chol monolayers, all*-trans* MK-7 exhibits in cyclic voltammetry a reversible redox system (Fig. 1). The mid-peak potentials of all*-trans* MK-7 are higher in the fluid phase, i.e., above the *T*_m, DMPC_, for pH 7.4 and pH 9. Since the ${{\rm p}}K_{{{\rm a}}}$ values of menaquinones are above 12 (Dharmaraj et al. 2020), this observation cannot be caused by the acidity of menaquinone, but it is obviously associated with the nature of the lipid phase. Measured in electrolytes of pH 4.0–12.0, the mid-peak potentials do not depend on the cholesterol content (0–35%). They are scattered within a 7 mV range (Table 1). This indicates that the thermodynamics of the redox system is not affected by cholesterol. However, the kinetics is affected (Table 2), as indicated by an increased peak separation at high cholesterol content. With the exception of pH 12.0, the high cholesterol content (35 mol %) in the DMPC films causes a slowdown of the kinetics of the all*-trans* MK-7 redox system. At that cholesterol content, DMPC is present as fluid lamellar ordered phase (L_α_ (o)). The peak separations are small when the DMPC exists as gel phase (G), G + L_α_ (o) and fluid lamellar disordered phase (L_α_ (d)) + L_α_ (o). There the peak separation is only a few mV, as typical for surface confined redox systems. The presence of cholesterol does not substantially affect the redox potentials of all*-trans* MK-7 system in DMPC/Chol films. Previously, a similar result has been reported by Becucci et al. (2011), who found that the thermodynamic redox potential of ubiquinone is not affected by the presence of cholesterol in dioleoylphosphatidylcholine–palmitoylsphingomyelin mixtures.

**Table 1:** Mid-peak potentials $E_{{{{\rm mp}}}}$ (versus Ag/AgCl (3 M KCl)) for DMPC/Chol films spiked with all*-trans* MK-7 for pH 4.0, 7.4, 9.0, and 12.0 at 20 °C and 28 °C.

pH	$E_{{{\rm{mp}}}}$[V vs. Ag/AgCl(3 M KCl)]								
	20 °C	28 °C	20 °C	28 °C	20 °C	28 °C	20 °C	28 °C	
	0 mol% Chol		5 mol% Chol		20 mol% Chol		35 mol% Chol		
4.0	− 0.109	− 0.107	− 0.099	− 0.104	− 0.104	− 0.109	− 0.094	− 0.109	
7.4	− 0.307	− 0.314	− 0.299	− 0.313	− 0.303	− 0.313	− 0.304	− 0.319	
9.0	− 0.409	− 0.419	− 0.398	− 0.419	− 0.401	− 0.413	− 0.407	− 0.416	
12.0	− 0.580	− 0.579	− 0.575	− 0.570	− 0.576	− 0.578	− 0.577	− 0.582	

At least three different monolayers were studied for each mid-peak potentials determination. Scan rate: 10 mV s^−1^.

**Table 2:** Separation of anodic and cathodic peaks for DMPC/Chol films spiked with all*-trans* MK-7. Scan rate: 10 mV s^−1^.

pH	$\Delta E_{{\rm{pa/pc}}}$[mV]							
	20 °C	28 °C	20 °C	28 °C	20 °C	28 °C	20 °C	28 °C
	0 mol% Chol		5 mol% Chol		20 mol% Chol		35 mol% Chol	
4.0	8 (± 2)	10 (± 4)	8 (± 2)	1 (± 0)	18 (± 4)	13 (± 2)	65 (± 25)	55 (± 11)
7.4	3 (± 1)	7 (± 1)	5 (± 2)	8 (± 2)	5 (± 1)	8 (± 2)	43 (± 3)	44 (± 23)
9.0	6 (± 4)	3 (± 2)	3 (± 3)	9 (± 2)	5 (± 2)	3 (± 1)	89 (± 32)	13 (± 4)
12.0	2 (± 1)	0 (± 0)	2 (± 1)	3 (± 1)	1 (± 1)	3 (± 0)	5 (± 1)	3 (± 0)

At least 3 different monolayers were studied for each $\Delta E_{{\rm{pa/pc}}}$ determination. In brackets, the standard deviations are given

### Kinetics of the electrochemical redox reactions of menaquinones in DMPC/cholesterol monolayers

A commonly used method to access the electron transfer rate constants of adsorbed redox systems is the Laviron formalism (Laviron 1979, 1982). The apparent rate constants (*k*_app_) for peak separations, $\Delta E_{{\rm{pa/pc}}}$ < 200 mV/*n* and $\Delta E_{{\rm{pa/pc}}}$ > 200 mV/*n* are determined according to the Laviron formalism. For the non-reversible case, where $\Delta E_{{\rm{pa/pc}}}$ > 200 mV/*n*, the following equations have to be used:

1 $$E_{{{\rm{pc}}}} = {E}_{\rm c}^{\ominus{^\prime}} - \left( {\frac{2.3RT}{{\alpha nF}}} \right){\rm{log}}\left[ {\frac{{\alpha nF\upsilon_{{\rm{c}}} }}{{RTk_{{{\rm{app}}}} }}} \right],$$

2 $$E_{{{\rm{pa}}}} = {E}_{\rm c}^{\ominus{^\prime}} - \left( {\frac{2.3RT}{{(1 - \alpha )nF}}} \right){\rm{log}}\left[ {\frac{{{(1} - \alpha )nF\upsilon_{{\rm{a}}} }}{{RTk_{{{\rm{app}}}} }}} \right],$$

3 $$k_{{{\rm{app}}}} = \frac{{\alpha nF\upsilon_{{\rm{c}}} }}{RT} = \frac{{(1 - \alpha )nF\upsilon_{{\rm{a}}} }}{RT}.$$

The critical scan rates $\upsilon_{{\rm{a}}}$ and $\upsilon_{{\rm{c}}}$ are obtained by plotting $E_{{\rm{pc(pa)}}} - {E}_{\rm c}^{\ominus{^\prime}} {\rm{ vs}}{\rm{. log }}\upsilon$, and extrapolating the slopes to $E_{{\rm{pc(pa)}}} - {E}_{\rm c}^{\ominus{^\prime}} = 0$, i.e., the *x*-intercept, where $E_{{\rm{pc(pa)}}}$ are the cathodic and anodic peak potentials, respectively, and ${E}_{\rm c}^{\ominus{^\prime}}$ is the formal (or mid-peak) potential. The values of $\alpha n$ and $(1 - \alpha )n$ are calculated from the slopes of $E_{{\rm{pc(pa)}}} - {E}_{\rm c}^{\ominus{^\prime}} {\rm{ vs}}{\rm{. log }}\upsilon$ where the slope is $- 2.3\frac{RT}{{\alpha nF}}$ for the cathodic branch and $2.3\frac{RT}{{(1 - \alpha )nF}}$ for the anodic branch, respectively. The rate constants are calculated for both critical scan rates and the mean values are given here. For the reversible and quasi-reversible cases, where $\Delta E_{{\rm{pa/pc}}}$ < 200 mV/*n*, the value of $\alpha$ for different temperatures was found by relating the ratio $y{ = }\left| {\frac{{E_{{{\rm{pc}}}} - {E}_{\rm c}^{\ominus{^\prime}} }}{{E_{{{\rm{pa}}}} - {E}_{\rm c}^{\ominus{^\prime}} }}} \right|$ to $\Delta E_{{\rm{pa/pc}}}$. Since *y* was equal to 1, $\alpha$ is 0.5, independent of the peak separations. The rate constants for different temperatures are determined from the plot of $\Delta E_{{\rm{pa/pc}}}$ < 200 mV/*n* vs 1/*m* for $\alpha = 0.5$, where $\frac{1}{m} = \frac{nF\upsilon }{{RTk_{{{\rm{app}}}} }}$. For different scan rates, $k_{{{\rm{app}}}}$ is calculated and the mean values are reported. There might be small errors in $k_{{{\rm{app}}}}$ values because the Laviron method is available only for 25 °C.

**Fig. 2:**
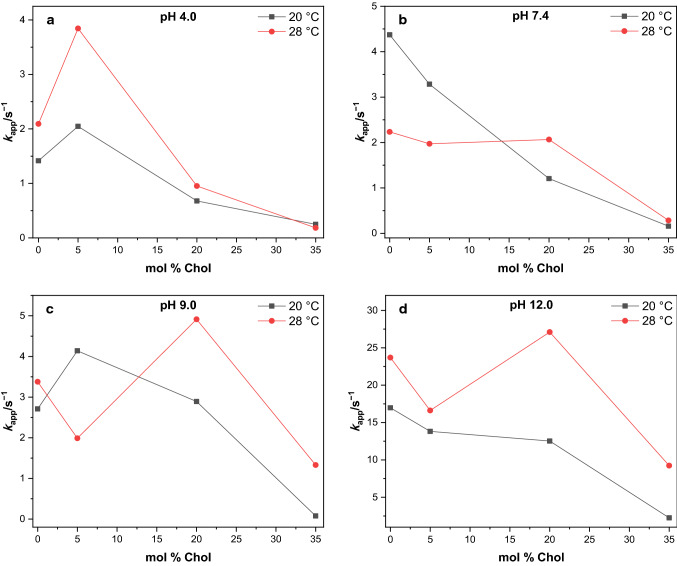
Dependence of apparent electron transfer rate constants of all*-trans* MK-7 on cholesterol content in DMPC films at 20 °C and 28 °C for (**a**) pH 4.0, (**b**) pH 7.4, (**c**) pH 9.0, and (**d**) pH 12.0

Using the Laviron formalism, the electron transfer rate constants of all*-trans* MK-7 in DMPC/Chol films were calculated at above and below the *T*_m,DMPC_ (Fig. 2, Table S1). The $k_{{{\rm{app}}}}$ data do not follow any specific dependence; rather several cases are observed:

i. The $k_{{{\rm{app}}}}$ of all*-trans* MK-7 in L_α_ (d) + L_α_ (o) phase (above the *T*_m, DMPC_) is higher than in the G + L_α_ (o) phase for all pH.
ii. In the G phase, the $k_{{{\rm{app}}}}$ of all*-trans* MK-7 increases with increasing pH, but in the (L_α_ (d)) phase, the $k_{{{\rm{app}}}}$ of all*-trans* MK-7 is almost constant at pH 7.4 and 9.0 which is also lower than the value at pH 4.0.
iii. Even in the (L_α_ (o)) phase, two different cases are observed at 20 °C and 28 °C: at 28 °C, $k_{{{\rm{app}}}}$ increases with decreasing in proton activity, and at 20 °C, the rate constants decline with decreasing proton activity (with the exception of pH 12.0).
iv. Generally in all phases, $k_{{{\rm{app}}}}$ is larger in the alkaline solution (pH 12.0).

The reason for the complex dependence of the $k_{{{\rm{app}}}}$ of all*-trans* MK-7 on cholesterol content might be the presence of different structural phases. The presence of cholesterol disturbs the order of the lipids, fluidity of the monolayer, reduces the surface area per lipid and causes a phase separation (domains or rafts) (Hernández et al. 2008a, 2008b). The presence of domains and the changes in the organization of the lipids can affect the all*-trans* MK-7 molecules for the electron transfer and accessibility of the protons. Figure 2 clearly indicates that large cholesterol concentrations decrease the rate constant.

In Fig. S2, the apparent electron transfer rate constants of MK-7 in DMPC/Chol monolayers are given as function of pH at temperatures above and below the phase transition temperature of DMPC. In all cases, the rate constants increase considerably in the alkaline range, i.e., in a clearly non-physiological range. See further down a completely different pH behavior in case of monolayers of natural cardiolipins.

### Effects of an inert salt (sodium perchlorate) addition to the aqueous phase on the thermodynamics and kinetics of the electrochemistry of all-trans MK-7 in DMPC monolayers

The inner of cells and mitochondria is by far no diluted aqueous solution, but a rather concentrated, quasi-crystalline solution of proteins and salts. Therefore, it is desirable to study not only the effects of membrane composition on the electrochemistry of menaquinones, but also the effects of composition of the aqueous phase. Hence, experiments have been performed in which an inert salt (sodium perchlorate) has been added to the aqueous buffer phase. The addition of this salt results at least in the following three alterations: (i) it changes the ionic strength (see Table 3). (ii) It changes the water activity. In 6 m (molal) solutions of NaClO_4_, water activity decreases to about 0.8 (Toner et al. 2016). (iii) The salt addition also diminishes the diffusion coefficient of protons (Roberts et al. 1974), which may affect the kinetics of the 2e^−^/2H^+^ redox reaction of the naphthoquinone unit. The inert salt also affects the pH of the buffer solutions, but that effect has been taken into consideration as follows: the pH of the solutions with salt additions has been measured and the mid-peak potential of the all*-trans* MK-7 of these solutions has been compared with that of NaClO_4_-free solutions of the respective pH values. To study the salt effect, the concentration of sodium perchlorate has been varied from 0 up to 5 mol kg^−1^, in addition to the used buffers (see experimental part). The measured potential differences $\Delta {E^{\prime}} = E_{{\rm{mp, exp}}} - E_{{\rm{mp, theoretical at given pH}}}$ are given in Table 3.

**Table 3:** DMPC films spiked with all*-trans* MK-7: $\Delta {E^{\prime}} = E_{{\rm{mp, exp}}} - E_{{\rm{mp, theoretical at given pH}}}$: Difference between experimentally measured mid-peak potentials and those at the measured pH, but without sodium perchlorate.

NaClO_4_ [m]	pH	*I* [mol kg^−1^]	$\Delta {E^{\prime}}$[V]	$\Delta E_{{\rm{pa/pc}}}$[V]
0.0	7.36	0.259	0	0.008 (± 0.003)
0.1	7.22	0.359	0.001	0.015 (± 0.001)
1.0	6.82	1.259	0.013	0.013 (± 0.001)
3.0	6.41	3.259	0.029	0.015 (± 0.005)
5.0	6.23	5.259	0.022	0.013 (± 0.003)

$\Delta E_{{\rm{pa/pc}}}$ is the peak separation between anodic and cathodic peaks. The film composition was 300 μmol DMPC + 5 μmol MK-7

Clearly, the effect of sodium perchlorate addition to the aqueous phase on the mid-peak potentials, i.e., on thermodynamics, is not negligible but small (1–29 mV). The effect on kinetics (anodic–cathodic peak separation) is, if at all, also very small (cf. Table 3)

### Thermodynamics of the electrochemistry of menaquinones in cardiolipin monolayers on mercury

Since cardiolipins are major constituents of mitochondrial membranes, the electrochemistry of menaquinones has been interrogated in monolayers of an artificial cardiolipin (TMCL) and in monolayers of natural cardiolipin (nCL).

**Fig. 3:**
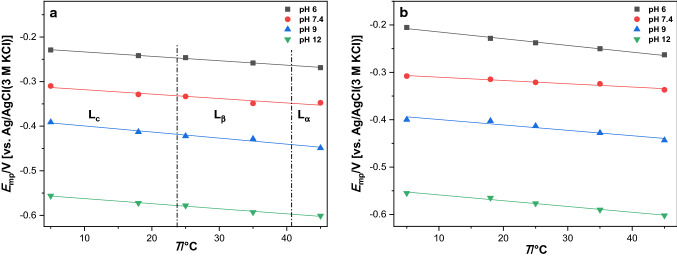
Dependence of mid-peak potentials of all*-trans* MK-4 spiked in (**a**) TMCL and (**b**) nCL films on temperature. Scan rate: 10 mV s^−1^. The dash dotted lines in TMCL/all*-trans* MK-4 film represent the phase transition temperatures. The ratio of all*-trans* MK-4 to TMCL was 2.2 µmol to 130.0 µmol TMCL (nCL, respectively)

The mid-peak potentials of all*-trans* MK-4 in TMCL and nCL monolayers continuously shift in the negative direction with increasing temperature (5–45 °C). There is no indication that the phase transitions of TMCL affect the potential shift (cf. Fig. 3). The temperature dependence of the mid-peak potentials allows calculating the reaction entropy $\Delta S$ given by

4 $$\Delta S = nF\left( {\frac{{{\rm{d}}E_{{{\rm{midpeak}}}} }}{{{\rm{d}}T}}} \right) = S_{{{\rm{MQH}}_{{2}} }} - S_{{{\rm{MQ}}}} ,$$

where $\left( {\frac{{{\rm{d}}E_{{{\rm{midpeak}}}} }}{{{\rm{d}}T}}} \right)$ is the slope in the plot of *E*_midpeak_ vs *T*.

Table 4 shows the reaction entropies of all*-trans* MK-4 in TMCL and nCL films.

**Table 4:** Reaction entropies of all*-trans* MK-4 in TMCL and nCL films. The ratio of all*-trans* MK-4 to TMCL was 2.2 µmol to 130.0 µmol TMCL (nCL, respectively)

pH	$\Delta S_{{\rm{TMCL/MK - 4}}}$[J K^−1^ mol^−1^]	$\Delta S_{{\rm{nCL/MK - 4}}}$[J K^−1^ mol^−1^]
6.0	− 191 (± 8)	− 274 (± 14)
7.4	− 191 (± 33)	− 133 (± 16)
9.0	− 262 (± 24)	− 220 (± 37)
12.0	− 220 (± 11)	− 235 (± 16)

Since these entropies refer to the reduction of the naphthoquinone to the naphthohydroquinone moiety

$${\rm{R}} - {\rm{NQ + 2 e}}^{ - } {\rm{ + 2H}}^{ + } \to {\rm{R}} - {\rm{NQH}}_{2}$$

it involves the dehydration of the protons, which is known to *increase* the entropy by + 131 J K^−1^ mol^−1^ (Marcus 2015). For the reduction of tetrafluoroquinone (TFQ) dissolved in aqueous solution, Yousoufian (Yousofian-Varzaneh et al. 2015) determined a *loss* of entropy of − 3.665 kJ K^−1^ mol^−1^, and they assumed as reason the decrease of number of particles during the reduction ${\rm{TFQ + 2 e}}^{ - } {\rm{ + 2H}}^{ + } \to {\rm{TFQH}}_{2}$. Wass et al. (Johnsson Wass et al. 2006) performed a quantum chemical modeling of the reduction of some quinones, including p-naphthoquinone to *cis*- and *trans*-naphthohydroquinone. They have found the following data for the reduction of p-naphthoquinone to the more stable *cis*-naphthohydroquinone: $\Delta G^{\bigcirc } = - 50.0{\rm{ kJ mol}}^{ - 1}$, $\Delta H^{\bigcirc } = - 86.0{\rm{ kJ mol}}^{ - 1}$ and $\Delta S^{\bigcirc } = - 121{\rm{ J K}}^{ - 1} {\rm{ mol}}^{ - 1}$. These data are not in contradiction to the experimental data, which we report here for all*-trans* MK-4 in TMCL and nCL films (Tables 5, 6, 7). However, it is interesting that the entropy loss is in case of the immobilized menaquinones much larger than in case of dissolved naphthoquinone. This may indicate a strong ordering of the menaquinone environment in the monolayer upon reduction.

**Table 5:** Thermodynamic parameters for the all*-trans* MK-4 redox couple MQ/MQH_2_ in TMCL and nCL films at pH = 0

$\Delta S_{{\rm{pH = 0}}}$ [J K^−1^ mol^−1^]		*T* [K]	${\Delta }G_{{\rm{pH = 0}}} { = } - nFE_{{{\rm{mp}}}}$^*^ [kJ mol^−1^]		${\Delta }H_{{\rm{pH = 0}}} {{ = \Delta }}G_{{\rm{pH = 0}}} + T\Delta S$ [kJ mol^−1^]	
TMCL + MK-4	nCL + MK-4		TMCL + MK-4	nCL + MK-4	TMCL + MK-4	nCL + MK-4
− 161.60	− 208.41	278.15	− 58.22	− 64.31	− 103.17	− 122.28
		291.15	− 55.51	− 59.58	− 102.56	− 120.26
		298.15	− 54.87	− 58.72	− 103.05	− 120.85
		308.15	− 52.77	− 57.86	− 102.57	− 122.08
		318.15	− 51.80	− 55.22	− 103.22	− 121.53

^*^$$E_{{{\rm{mp}}}}$$ vs SHE

**Table 6:** Thermodynamic parameters for the all*-trans* MK-4 redox couple MQ/MQH_2_ in TMCL films

TMCL/MK-4												
	${\Delta }G{ = } - nFE_{{{\rm{mp}}}}$* [kJ mol^−1^]				$T\Delta S$ [kJ mol^−1^]				${\Delta }H{{ = \Delta }}G + T\Delta S$ [kJ mol^−1^]			
*T* [K]	pH 6.0	pH 7.4	pH 9.0	pH 12.0	pH 6.0	pH 7.4	pH 9.0	pH 12.0	pH 6.0	pH 7.4	pH 9.0	pH 12.0
278.15	4.27	19.86	35.52	67.39	− 53.13	− 53.20	− 73.00	− 61.19	− 48.86	− 33.34	− 37.48	6.20
291.15	6.74	23.49	39.73	70.51	− 55.61	− 55.68	− 76.41	− 64.05	− 48.87	− 32.19	− 36.68	6.46
298.15	7.59	24.39	41.53	71.62	− 56.95	− 57.02	− 78.25	− 65.59	− 49.35	− 32.64	− 36.71	6.03
308.15	9.91	27.39	42.75	74.49	− 58.86	− 58.94	− 80.87	− 67.79	− 48.94	− 31.54	− 38.12	6.70
318.15	11.93	27.11	46.68	76.04	− 60.77	− 60.85	− 83.50	− 69.99	− 48.84	− 33.74	− 36.81	6.05

^*^$$E_{{{\rm{mp}}}}$$ vs SHE

**Table 7:** Thermodynamic parameters for the all*-trans* MK-4 redox couple MQ/MQH_2_ in nCL films

nCL/MK-4												
	${\Delta }G{ = } - nFE_{{{\rm{mp}}}}$* [kJ mol^−1^]				$T\Delta S$[kJ mol^−1^]				${\Delta }H{{ = \Delta }}G + T\Delta S$[kJ mol^−1^]			
*T* [K]	pH 6.0	pH 7.4	pH 9.0	pH 12.0	pH 6.0	pH 7.4	pH 9.0	pH 12.0	pH 6.0	pH 7.4	pH 9.0	pH 12.0
278.15	− 0.30	19.50	37.22	67.13	− 76.22	− 37.06	− 61.19	− 65.48	− 76.52	− 17.56	− 23.97	1.65
291.15	4.12	20.79	37.71	69.07	− 79.78	− 38.79	− 64.05	− 68.54	− 75.66	− 18.00	− 26.34	0.53
298.15	5.92	22.03	39.80	71.30	− 81.70	− 39.72	− 65.59	− 70.19	− 75.78	− 17.69	− 25.79	1.11
308.15	8.34	22.64	42.61	73.89	− 84.44	− 41.06	− 67.79	− 72.55	− 76.10	− 18.42	− 25.18	1.34
318.15	10.82	25.07	45.61	76.24	− 87.18	− 42.39	− 69.99	− 74.90	− 76.36	− 17.32	− 24.38	1.34

^*^$$E_{{{\rm{mp}}}}$$ vs SHE

Since the addition of 2.2 µmole all*-trans* MK-4 to TMCL has practically no effect on the phase transition temperatures of TMCL (40.7 °C and 23.8 °C) (Fig. S1) determined previously in a chronoamperometry study (Zander et al. 2012), it can be assumed that the two components do not form specific phases, and further, that the menaquinone does not alter the TMCL phases. Natural cardiolipins (nCL) and nCL containing all*-trans* MK-4 liposomes do not exhibit any phase transitions in the temperature range 7 to 90 °C. Because all*-trans* MK-4 has no effect on the TMCL phases, it is reasonable to assume that all*-trans* MK-4 forms also in nCL just a diluted solution.

**Fig. 4:**
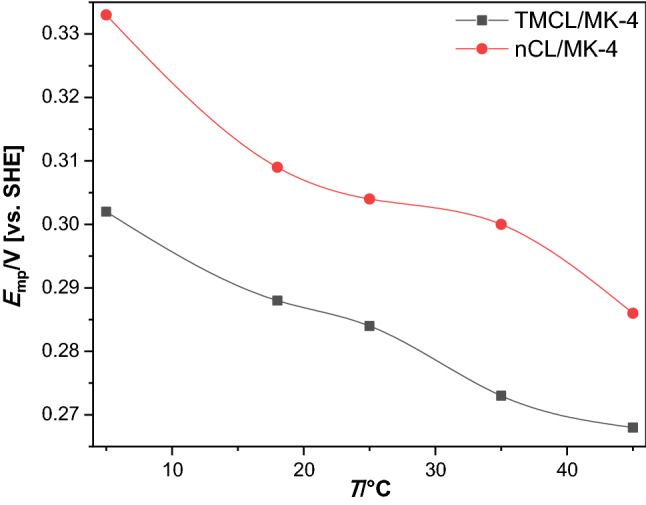
Redox potentials of all*-trans* MK-4 in TMCL ($E^{\ominus^{\prime}}_{\rm{TMCL/MK - 4}}$) and in nCL ($E^{\ominus^{\prime}}_{\rm{nCL/MK - 4}}$) films at different temperatures. Scan rate: 10 mV s^−1^. The ratio of all*-trans* MK-4 to TMCL was 2.2 µmol to 130.0 µmol TMCL (nCL, respectively)

The TMCL/all*-trans* MK-4 and nCL/all*-trans* MK-4 exhibit slow electron transfer kinetics and the quantitative evaluation was performed using the Laviron formalism (see below). The separation of anodic and cathodic peak potentials decreases considerably with increasing temperature (Figs. S3 to S6). In case of TMCL, the different phases exhibit different slopes of peak separation and peak potentials versus temperature. This clearly indicates that the nature of the phases affects the kinetics. The formal potential ($E_{{{\rm{MQ/MQH}}_{{2}} }}^{\ominus{^\prime}}$) of the MQ/MQH_2_ couple for different temperatures are easily obtained from the dependence of $E_{{{\rm{mp}}}}$ on pH by extrapolating to the unitary proton activity (pH = 0) and the slopes obey linear dependences (Fig. S7, Table S2) between pH 6.0 and 12.0. All*-trans* MK-4 shows in nCL films higher redox potentials than in TMCL films (cf. Fig. 4). Thus, the nature of the lipids housing the all*-trans* MK-4 determines the redox potential, which is highly important to understand the biochemical reactions, notably in biological membranes.

### Kinetics of the electrochemistry of menaquinones in cardiolipin monolayers on mercury

**Fig. 5:**
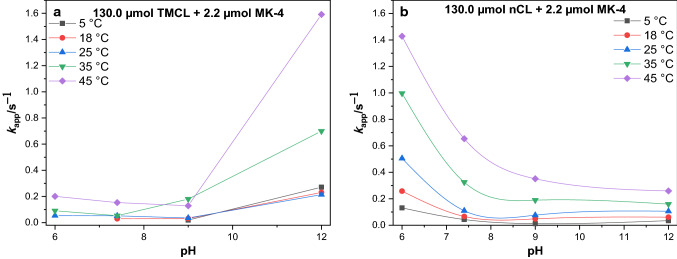
Apparent electron transfer rate constants of MK-4 in (**a**) TMCL and (**b**) nCL films in dependence on pH. The ratio of all*-trans* MK-4 to TMCL was 2.2 µmol to 130.0 µmol TMCL (nCL, respectively)

**Fig. 6:**
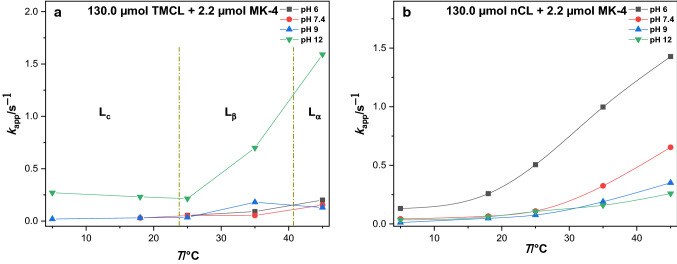
Dependence of rate constants $k_{{{\rm{app}}}}$ of all*-trans* MK-4 in (**a**) TMCL and (**b**) nCL films on temperatures. The ratio of all*-trans* MK-4 to TMCL was 2.2 µmol to 130.0 µmol TMCL (nCL, respectively)

The apparent electron transfer coefficient, $\alpha$ of all*-trans* MK-4 in TMCL and nCL films was determined (Table S3). For $\Delta E_{{\rm{p}}}$ > 200/*n* mV, the mean value of anodic and cathodic $\alpha$ is around 0.5 which agrees with *n* = 2. For the quasi- and completely reversible system, where $\Delta E_{{\rm{pa/pc}}}$ < 200/*n* mV, $\alpha$ is 0.5 (Laviron formalism). The $k_{{{\rm{app}}}}$ of all*-trans* MK-4 in TMCL and nCL was estimated using the Laviron method (see rate constants determination section). For nCL/all*-trans* MK-4 films, $k_{{{\rm{app}}}}$ is always highest at pH 6.0, given that only the pH range of 6.0–12.0 has been studied. Most interestingly, in contrast to these results, all*-trans* MK-4 in TMCL exhibits highest $k_{{{\rm{app}}}}$ values at pH 12.0 (Figs. 5, 6, and Table S4).

**Fig. 7:**
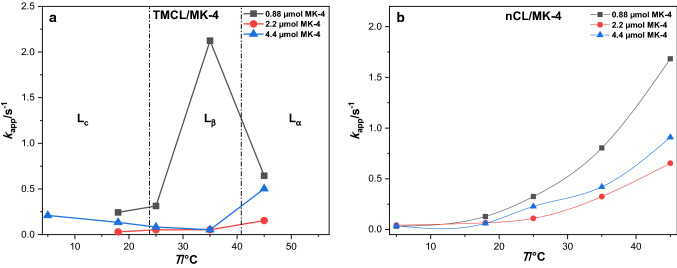
Dependence of rate constants of all*-trans* MK-4 concentrations (0.88 µmol, 2.2 µmol, 4.4 µmol per 130.0 µmol TMCL (nCL, respectively) in (**a**) TMCL and (**b**) nCL films on temperatures for pH 7.4

Looking at the dependence of $k_{{{\rm{app}}}}$ on the concentration of all*-trans* MK-4 in the films, in TMCL as well as in nCL, decreasing amounts of all*-trans* MK-4 give larger rate constants (cf. Fig. 7). Indeed, also in case of ubiquinone-10 monolayers, the maximum electron transfer rate constants have been found at lowest surface concentration (Sek et al. 1999). The rate constants $k_{{{\rm{app}}}}$ generally increase with increasing temperature for each concentration of all*-trans* MK-4 in TMCL and nCL films (Fig. 7). In TMCL films, the $k_{{{\rm{app}}}}$ of 4.4 µmol all*-trans* MK-4 slightly decreases in L_c_ and L_β_ phases, and increased in L_α_ phase. There is also an abruptly high $k_{{{\rm{app}}}}$ for the lowest all*-trans* MK-4 content (0.88 µmol) in the L_β_ phase. The correctness of this result is support by 3 independent film preparations and measurements.

Using the apparent electron transfer constants at different temperatures, the apparent activation energies are obtained using the Arrhenius Eq. (5) for TMCL/MK-4 and nCL/MK-4 films (Table 8):

5 $$\ln k_{{{\rm{app}}}} = \frac{{ - E_{{{\rm{act}}}} }}{R}\left( \frac{1}{T} \right) + \ln A,$$

where $E_{{{\rm{act}}}}$ is the apparent activation energy, *A* the Arrhenius constant, $k_{{{\rm{app}}}}$ the apparent electron transfer rate constant, *R* the gas constant, and *T* the temperature in Kelvin.

**Table 8:** Apparent activation energies of all*-trans* MK-4 spiked in TMCL and nCL films for pH = 6.0, pH = 7.4, pH = 9.0, and pH = 12.0.

pH	$E_{{{\rm{act}}}}$, _TMCL/MK-4_ [eV]	$E_{{{\rm{act}}}}$, _nCL/MK-4_ [eV]
6.0	0.53 (± 0.08)	0.48 (± 0.03)
7.4	0.43 (± 0.12)	0.54 (± 0.08)
9.0	0.43 (± 0.12)	0.64 (± 0.02)
12.0	0.82 (± 0.07) [*T* ≥ 298.15 K] − 0.08 (± 0.00) [*T* ≤ 298.15 K]	0.39 (± 0.02)

The ratio of all-*trans* MK-4 to TMCL was 2.2 µmol to 130.0 µmol TMCL (nCL, respectively)

For films of hydroquinone covalently bonded to PEDOT, Sterby et al. (Sterby et al. 2019) found an activation energy of 0.3 eV for the electrochemical redox reaction. Samuelson and Sharp (Samuelsson et al. 1978) determined the activation energies for 1,4-benzoquinone, 1,4-naphthoquinone and for 9,10-anthraquinone in acetonitrile solutions at Pt, Au and graphite electrodes to be all around 0.23 eV. The higher, but still very similar, values found for all*-trans* MK-4 can be easily explained with the long chain of the menaquinone-4 (4 isoprenoyl units, i.e., 16 carbon atoms in the chain, and 4 double bonds interconnected by 2 sp^3^ hybridized carbons). These chains are rather long and because of the sp^3^ hybridized carbons, they are obviously rather bad conductors for electrons, which explains the slower redox kinetics.

## Conclusion

The thermodynamics and kinetics of electrochemistry of menaquinones have been studied using lipid monolayers on mercury. These are the conclusions:

i. There is no significant effect of cholesterol when added to the films on the thermodynamics of all*-trans* MK-7 in DMPC films, but the kinetics of the electrochemistry of all*-trans* MK-7 is affected at high cholesterol content. The electron transfer rate constants depend on the DMPC phases and the pH. The fact that the thermodynamics of the electrochemistry of all*-trans* MK-7 in DMPC films is not affected by the presence of cholesterol indicates that the latter does not interact directly with the menaquinone in the film. The effect of cholesterol on the kinetics may result from a changed double-layer structure at the solution|film interface.
ii. There is a slight increase of the thermodynamic mid-peak potentials of all*-trans* MK-7 in DMPC films on lowering the water activity by increasing inert salt concentration (ionic strength) in the aqueous phase. The effect is small, but not negligible. The water activity (ionic strength) has practically no effect on the kinetics of the electrochemistry of all*-trans* MK-7.
iii. The addition of all*-trans* MK-4 to TMCL does not change the phase transitions of TMCL. The changes in reaction entropy, enthalpy and free energy, and activation energies were determined for all*-trans* MK-4 in TMCL and nCL films. The nature of the lipids affects the redox potential of all*-trans* MK-4. The electron transfer rate constant of all*-trans* MK-4 is affected by the type of lipids, the nature of lipid phases, the temperature, and the amount of all*-trans* MK-4.
iv. The pH dependence of rate constants of all*-trans* MK-4 in TMCL and nCL films are completely opposite. This is most interesting and indicates that natural cardiolipins have obviously very special properties for redox reactions of incorporated redox species. It may not be accidental that natural cardiolipins provide high rate constants of redox cycling at physiological pH and temperature.

The investigations reported in this work emphasize that the environment of redox systems in membranes is important for their thermodynamics and kinetics. Therefore, elucidating the quantitative function of electron shuttling molecules in membranes needs model systems which include all constituents of membranes. Unfortunately, here we could not include membrane bound proteins, which have to be included in future studies.

## Supplementary Information

Below is the link to the electronic supplementary material.

Supplementary file1 (DOCX 2916 KB)

## Abbreviations

*α*: Electron transfer coefficient
*E*_act_: Activation energy
$${E}_{\text {c}}^{\ominus{^\prime}}$$: Formal potential
$$E_{{{{\rm {mp}}}}}$$: Midpeak potential
$E_{{{{\rm {pa}}}}}$: Anodic peak potential
$E_{{{{\rm {pc}}}}}$: Cathodic peak potential
*E*_pc(pa)_: Cathodic peak potential or anodic peak potential
∆*E*ʹ: $$ E_{{{\rm {mp}, \rm {exp}}}} - E_{{{\rm {mp}, \rm {theoretical at given pH}}}}$$
$\Delta E_{{{pa/pc}}}$: Peak separation between anodic and cathodic peaks
$F$: Faraday constant (96,485.3 C mol^−^^1^)
$k_{{{{app}}}}$: Apparent electron transfer rate constant
$R$: Gas constant (8.3145 J mol^−^^1^ K^−^^1^)
$\upsilon_{a}$: Critical anodic scan rate
$\upsilon_{c}$: Critical cathodic scan rate
$\Delta G^{\bigcirc }$: Standard free energy change
${\Delta }G$: Free energy change
$\Delta H^{\bigcirc }$: Standard enthalpy change
$${\Delta }H$$: Enthalpy change
$\Delta S^{\bigcirc }$: Standard entropy change
$$\Delta S$$: Entropy change
Chol: Cholesterol
CL: Cardiolipin
DMPC: (14:0 PC) 1,2-dimyristoyl-sn-glycero-3-phosphocholine
DMPC/Chol: DMPC lipids films containing Chol at different mol %
DMPC/Chol/*all-trans* MK-7: *all-trans* Menaquinone-7 in DMPC/Chol films
*G*: Gel phase of DMPC/Chol mixtures
*I*: Ionic strength of the solution
L_α_: Lamellar liquid crystalline phase of TMCL
L_β_: Lamellar gel phase of TMCL
L_c_: Subgel phase of TMCL
L_α_ (d): Fluid lamellar disordered phase of DMPC/Chol mixtures
L_α_ (o): Fluid lamellar ordered phase of DMPC/Chol mixtures
nCL: Natural cardiolipin (Heart, Bovine) (sodium salt)
TMCL: (14:0 Cardiolipin (sodium salt)) 1′,3′-bis[1,2-dimyristoyl-sn-glycero-3-phospho]-glycerol (sodium salt)
*T*_m, DMPC_: Phase transition temperature of DMPC
TMCL/all-*trans* MK-4: *all-trans* Menaquinone-4 in TMCL films
nCL/all-*trans* MK-4: *all-trans* Menaquinone-4 in nCL films
