# Supplementary material for: The effects of the chemical environment of menaquinones in lipid monolayers on mercury electrodes on the thermodynamics and kinetics of their electrochemistry
Source: Eur Biophys J. 2021 Mar 17;50(5):731–43. doi: 10.1007/s00249-021-01512-w (PMC8260536; doi:10.1007/s00249-021-01512-w)
Supplement: Supplementary file 1 — Supplementary file1 (DOCX 2916 KB) [file 249_2021_1512_MOESM1_ESM.docx]

**Supporting information**

**The effects of the chemical environment of menaquinones in lipid monolayers on mercury electrodes on the thermodynamics and kinetics of their electrochemistry.**

**Karuppasamy Dharmaraj^1^, Dirk Dattler^1^, Heike Kahlert^1^, Uwe Lendeckel^2^, Felix Nagel^1^, Mihaela Delcea^1^, Fritz Scholz^1^**

**E-mail:** fscholz@uni-greifswald.de

ORCID of the authors:

Prof. Dr. Fritz Scholz: 0000-0001-6287-1184

Karuppasamy Dharmaraj: 0000-0001-6743-3503
PD Dr. Heike Kahlert: 0000-0002-9196-5750
Prof. Dr. Mihaela Delcea: 0000-0002-0851-9072
Prof. Dr. Uwe Lendeckel: 0000-0002-0684-9959

Dirk Dattler: 0000-0002-0139-588X

Felix Nagel: 0000-0003-3456-7075

1 Institute of Biochemistry, University of Greifswald, Felix-Hausdorff-Str. 4, 17487 Greifswald, Germany.

2 Institute of Medical Biochemistry and Molecular Biology, University Medicine Greifswald, University of Greifswald, Ferdinand-Sauerbruch-Str., D-17475 Greifswald, Germany.

Fig. S1 Differential scanning calorimetry (DSC) heating thermograms of (a) TMCL and TMCL/ *all-trans* MK-4 and (b) nCL and nCL/ *all-trans* MK-4 liposomes in pH 7.4 buffer. The ratio of *all-trans* MK-4 to TMCL was 2.2 µmol to 130.0 µmol TMCL (nCL, respectively)

Fig. S2 Apparent electron transfer rate constants of MK-7 in DMPC/Chol monolayers as function of pH at above and below the phase transition temperature of DMPC. (a) 0 mol % Chol, (b) 5 mol % Chol, (c) 20 mol % Chol, and (d) 35 mol % Chol

Fig. S3 Peak separations between anodic and cathodic peaks dependence on temperatures of TMCL films spiked with MK-4 for (a) pH 6.0, (b) pH 7.4, (c) pH 9.0, and (d) pH 12.0. The dash dotted lines represent the phase transition temperatures. The film composition was 130.0 μmol TMCL + 2.2 μmol MK-4

Fig. S4 Peak separations between anodic and cathodic peaks dependence on temperatures of nCL films spiked with MK-4 for (a) pH 6.0, (b) pH 7.4, (c) pH 9.0, and (d) pH 12.0. The film composition was 130.0 μmol nCL + 2.2 μmol MK-4

Fig. S5 Anodic peak potentials and cathodic peak potentials dependence on temperatures of TMCL films spiked with MK-4 for (a) pH 6.0, (b) pH 7.4, (c) pH 9.0, and (d) pH 12.0. The dash dotted lines represent the phase transition temperatures. Scan rate: 10 mV s^−1^. The film composition was 130.0 μmol TMCL + 2.2 μmol MK-4

Fig. S6 Anodic peak potentials and cathodic peak potentials dependence on temperatures of nCL films spiked with MK-4 for (a) pH 6.0, (b) pH 7.4, (c) pH 9.0, and (d) pH 12.0. Scan rate: 10 mV s^−1^. The film composition was 130.0 μmol nCL + 2.2 μmol MK-4

Fig. S7 Dependence of mid-peak potentials of MK-4 in (a) TMCL and (b) nCL films on pH. Scan rate: 10 mV s^−1^. The ratio of all-trans MK-4 to TMCL was 2.2 µmol to 130.0 µmol TMCL (nCL, respectively)

Table S1 Apparent electron transfer rate constants of MK-7 in DMPC/Chol films above and below *T*_m, DMPC_ for pH 4.0, 7.4, 9.0, and 12.0

| pH 4.0 | 20 °C | 28 °C |
| --- | --- | --- |
| mol % Chol | $k_{\text{app}}$ [s^-1^] | $k_{\text{app}}$ [s^-1^] |
| 0 | 1.42 | 2.09 |
| 5 | 2.05 | 3.84 |
| 20 | 0.68 | 0.95 |
| 35 | 0.25 | 0.18 |

| pH 7.4 | 20 °C | 28 °C |
| --- | --- | --- |
| mol % Chol | $k_{\text{app}}$ [s^-1^] | $k_{\text{app}}$ [s^-1^] |
| 0 | 4.37 | 2.24 |
| 5 | 3.28 | 1.97 |
| 20 | 1.21 | 2.07 |
| 35 | 0.16 | 0.28 |

| pH 9.0 | 20 °C | 28 °C |
| --- | --- | --- |
| mol % Chol | $k_{\text{app}}$ [s^-1^] | $k_{\text{app}}$ [s^-1^] |
| 0 | 2.71 | 3.38 |
| 5 | 4.14 | 1.99 |
| 20 | 2.89 | 4.91 |
| 35 | 0.08 | 1.33 |

| pH 12.0 | 20 °C | 28 °C |
| --- | --- | --- |
| mole % Chol | $k_{\text{app}}$ [s^-1^] | $k_{\text{app}}$ [s^-1^] |
| 0 | 16.97 | 23.69 |
| 5 | 13.82 | 16.61 |
| 20 | 12.53 | 27.10 |
| 35 | 2.25 | 9.24 |

Table S2 Slopes of mid-peak potentials vs pH of TMCL and nCL films spiked with MK-4 in the pH range 6.0 to 12.0. The ratio of all-trans MK-4 to TMCL was 2.2 µmol to 130.0 µmol TMCL (nCL, respectively)

| *T* [°C] | TMCL/MK-4 | nCL/MK-4 |
| --- | --- | --- |
|  | Slopes [V/pH] | Slopes [V/pH] |
| 5 | −0.054 (± 0.001) | −0.057 (± 0.003) |
| 18 | −0.055 (± 0.001) | −0.056 (± 0.001) |
| 25 | −0.055 (± 0.002) | −0.056 (± 0.001) |
| 35 | −0.055 (± 0.002) | −0.057 (± 0.001) |
| 45 | −0.056 (± 0.002) | −0.057 (± 0.002) |

Table S3 Apparent electron transfer coefficients of MK-4 in TMCL and nCL films. The ratio of *all-trans* MK-4 to TMCL was 2.2 µmol to 130.0 µmol TMCL (nCL, respectively)

| TMCL/MK-4 | | | | | nCL/ MK-4 | | | | |
| --- | --- | --- | --- | --- | --- | --- | --- | --- | --- |
| pH | *T* [°C] | (1−*α*)*n* | *αn* | mean *α* (*n*=2) | pH | *T* [°C] | (1−*α*)*n* | *αn* | mean *α* (*n*=2) |
| 6.0 | 25 | 0.68 | 0.66 | 0.49 | 7.4 | 5 | 0.60 | 0.32 | 0.43 |
|  |  |  |  |  |  |  |  |  |  |
|  | 18 | 0.65 | 0.65 | 0.50 | 9.0 | 5 | 0.57 | 0.48 | 0.48 |
| 7.4 | 25 | 0.66 | 0.66 | 0.50 |  | 18 | 0.56 | 0.62 | 0.51 |
|  | 35 | 0.77 | 0.91 | 0.54 |  |  |  |  |  |
|  |  |  |  |  |  |  |  |  |  |
|  | 5 | 0.59 | 0.54 | 0.49 | 12.0 | 5 | 0.42 | 0.42 | 0.50 |
| 9.0 | 18 | 0.62 | 0.61 | 0.50 |  |  |  |  |  |
|  | 25 | 0.64 | 0.77 | 0.53 |  |  |  |  |  |

Table S4 Apparent electron transfer rate constants of MK-4 in TMCL and nCL films at different temperatures for pH 6.0, pH 7.4, pH 9.0, and pH 12.0. The ratio of *all-trans* MK-4 to TMCL was 2.2 µmol to 130.0 µmol TMCL (nCL, respectively)

| pH 6.0 |  |  |
| --- | --- | --- |
| *T* [°C] | TMCL/MK-4, $k_{\text{app}}$ [s^-1^] | nCL/MK-4, $k_{\text{app}}$ [s^-1^] |
| 5 |  | 0.13 |
| 18 |  | 0.26 |
| 25 | 0.05 | 0.51 |
| 35 | 0.09 | 1.00 |
| 45 | 0.20 | 1.43 |

| pH 7.4 |  |  |
| --- | --- | --- |
| *T* [°C] | TMCL/MK-4, $k_{\text{app}}$ [s^-1^] | nCL/MK-4, $k_{\text{app}}$ [s^-1^] |
| 5 |  | 0.04 |
| 18 | 0.03 | 0.07 |
| 25 | 0.05 | 0.11 |
| 35 | 0.05 | 0.33 |
| 45 | 0.15 | 0.65 |

| pH 9.0 |  |  |
| --- | --- | --- |
| *T* [°C] | TMCL/MK-4, $k_{\text{app}}$ [s^-1^] | nCL/MK-4, $k_{\text{app}}$ [s^-1^] |
| 5 | 0.02 | 0.01 |
| 18 | 0.03 | 0.05 |
| 25 | 0.04 | 0.08 |
| 35 | 0.18 | 0.19 |
| 45 | 0.13 | 0.35 |

| pH 12.0 |  |  |
| --- | --- | --- |
| *T* [°C] | TMCL/MK-4, $k_{\text{app}}$ [s^-1^] | nCL/MK-4, $k_{\text{app}}$ [s^-1^] |
| 5 | 0.27 | 0.03 |
| 18 | 0.23 | 0.06 |
| 25 | 0.21 | 0.11 |
| 35 | 0.70 | 0.16 |
| 45 | 1.59 | 0.26 |
